# Noncoding RNAs as emerging regulators of *Plasmodium falciparum* virulence gene expression

**DOI:** 10.1016/j.mib.2014.06.013

**Published:** 2014-08

**Authors:** Shruthi S Vembar, Artur Scherf, T Nicolai Siegel

**Affiliations:** 1 Biology of Host-Parasite Interactions Unit, Institut Pasteur, Paris, France; 2 CNRS URA2581, Paris, France; 3 Research Center for Infectious Diseases, University of Wuerzburg, Josef-Schneider-Str. 2/Bau D15, 97080 Wuerzburg, Germany

## Abstract

The eukaryotic unicellular pathogen *Plasmodium falciparum* tightly regulates gene expression, both during development and in adaptation to dynamic host environments. This regulation is evident in the mutually exclusive expression of members of clonally variant virulence multigene families. While epigenetic regulators have been selectively identified at active or repressed virulence genes, their specific recruitment remains a mystery. In recent years, noncoding RNAs (ncRNAs) have emerged as lynchpins of eukaryotic gene regulation; by binding to epigenetic regulators, they provide target specificity to otherwise non-specific enzyme complexes. Not surprisingly, there is great interest in understanding the role of ncRNA in *P. falciparum*, in particular, their contribution to the mutually exclusive expression of virulence genes. The current repertoire of *P. falciparum* ncRNAs includes, but is not limited to, subtelomeric ncRNAs, virulence gene-associated ncRNAs and natural antisense RNA transcripts. Continued improvement in high-throughput sequencing methods is sure to expand this repertoire. Here, we summarize recent advances in *P. falciparum* ncRNA biology, with an emphasis on ncRNA-mediated epigenetic modes of gene regulation.

## Highlights

• Small and long noncoding RNAs are abundant in *Plasmodium falciparum* blood stages.
• Subtelomeric and intronic ncRNAs may determine singular *var* gene choice.
• Natural antisense transcripts may regulate gene expression in an epigenetic manner.
• Adaptation of new technologies to *Plasmodium* is vital to studying ncRNA function.

**Current Opinion in Microbiology** 2014, **20**:153–161

This review comes from a themed issue on **Host–microbe interactions: parasites**

Edited by **Manoj Duraisingh** and **Nancy Guillén**

For a complete overview see the Issue and the Editorial

Available online 12th July 2014

**http://dx.doi.org/10.1016/j.mib.2014.06.013**

1369-5274/© 2014 The Authors. Published by Elsevier Ltd. This is an open access article under the CC BY license (http://creativecommons.org/licenses/by/3.0/).

## Introduction

The protozoan parasite *Plasmodium falciparum* causes the most lethal form of human malaria, killing ∼600 000 people annually [1]. Disease symptoms manifest during intra-erythrocytic development (IED) in the human host, when parasites divide multiple times in a 48 h cycle. The IED also generates a small proportion of male and female gametocytes that are taken up by the mosquito vector and are essential for disease transmission. Stage-specific transcriptome analyses using microarrays [2, 3, 4, 5, 6, 7, 8] and next-generation sequencing (NGS) of cDNA (RNA-seq) [9] revealed that *P. falciparum* gene expression occurs in a tightly coordinated cascade, both during IED and during sexual development in the mosquito host (see also the review by Voss *et al.*, in this issue). This is noteworthy given the paucity of specific transcription factors in the *P. falciparum* genome [10], and evokes the presence of other regulatory pathways in the parasite.

During development, and in response to a constantly changing host environment (host metabolic and nutritional status, body temperature, immune factors, *etc.*), *P. falciparum* gene expression is modulated at the transcriptional, post-transcriptional, translational, and post-translational levels. One of the best-studied examples is the epigenetically regulated transcription of clonally variant, subtelomeric multigene families such as *var*, *rifin* and *stevor* during IED [11, 12] (see also the review by Voss *et al.*, in this issue). Chromatin immunoprecipitation followed by microarray analysis (ChIP-on-chip) showed that the genomic loci of transcriptionally inactive members of these families are associated with histone H3 lysine 9 tri-methylation (H3K9me3), a hallmark of heterochromatin, but not the active member(s), which are marked with H3K9 acetylation and H3K4me2/3 [13, 14]. These histone modifications are maintained over many parasite generations indicating heritability and cellular memory [15]: they also confer transcriptional variability amongst genetically identical parasites [16]. Moreover, these findings raise important questions: What are the molecular factors that initiate and propagate the epigenome of *P. falciparum*? How does the parasite alter clonally variant gene expression in response to host cues? While some histone-modifying enzymes have been identified, including the sirtuins PfSir2A and PfSir2B [17, 18, 19] and the histone lysine methyl transferase PfKMT1 [20], how these enzymes are targeted to specific genomic loci remains unknown. An emerging hypothesis is that the transcription of noncoding RNAs (ncRNAs) may be central to the site-specific targeting of these histone-modifying enzymes.

As their name suggests, ncRNAs are RNA transcripts that are not translated into proteins. Excluding structural RNAs such as ribosomal RNAs, spliceosomal RNAs and transfer RNAs, ncRNAs can be broadly divided into small (<200 nt) and long ncRNAs (>200 nt; lncRNAs). In addition, ncRNAs complementary to endogenous transcripts and longer than 200 nt form a unique subset referred to as natural antisense transcripts (NATs). In eukaryotes, small RNA-induced post-transcriptional gene silencing *via* the RNA-interference (RNAi) pathway is the best-characterized regulatory role of ncRNAs; however, the *P. falciparum* genome lacks components of the canonical dicer-dependent RNAi pathway that generates this class of ncRNAs [10, 21]. In contrast, NATs and lncRNAs, which have been shown to regulate gene expression in organisms lacking functional RNAi machinery, have been identified in *P. falciparum* using serial analysis of gene expression (SAGE), northern blots, microarrays and most recently, strand-specific RNA-seq [22, 23, 24, 25, 26, 27, 28•, 29•, 30•, 31•, 32•, see Box 1]. In this review, we describe the recent advances in *P. falciparum* ncRNA biology, with an emphasis on the putative contributions of ncRNAs to maintaining the epigenome and modulating mutually exclusive *var* gene expression.

Box 1 **NGS-related technical challenges faced by P. falciparum researchers**

Commonly, the preparation of RNA or DNA for NGS includes a PCR amplification step, which introduces biases in the resulting libraries [79]; in particular, regions high in AT or GC content are generally underrepresented or lost. Given that the *P. falciparum* genome contains 80.7% A+T, with many intronic and intergenic regions being up to 95% A+T, it has been difficult to obtain high and uniform coverage of the genome. To overcome this problem, alternative methods such as amplification-free library preparation [79] and T7 RNA polymerase-based linear amplification [56] methods have been developed and have produced more uniform genome coverage, even across very AT-rich regions. Nonetheless, these methods have other drawbacks: amplification-free library preparations require large amounts of starting material, while T7 RNA polymerase-based linear amplifications result in a higher number of chimeric and duplicate reads than PCR-amplified libraries [80].

In contrast, a key improvement has come from the design of DNA polymerases with lower sequence bias. For example, libraries prepared using the KAPA Hifi DNA polymerase (KAPA Biosystems) showed higher and more even genomic coverage than those prepared using Phusion polymerase (New England Biolabs), and were comparable to amplification-free libraries [81, 82••]. For the AT-rich *P. falciparum* genome, the best results were obtained using the KAPA HiFi DNA polymerase, annealing and extension temperatures of 60 °C, and increased annealing and extension times. Independent of the high AT-content, transcripts from subtelomeric regions, conserved *var* introns, *var* exon 2, and GC-rich elements have been difficult to analyze and map to a unique genomic locus, due to their highly repetitive nature. However, with sequence read length constantly increasing — for example, the PacBio platform is currently capable of sequencing transcripts of up to 7 kb in length — analysis of highly repetitive regions is now possible [83].

The discovery of antisense transcription in the *P. falciparum* genome (discussed in the Natural antisense RNA transcripts section) also requires library preparation protocols with a high degree of strand-specificity. Because reverse transcriptases can use cDNA as a template, earlier studies may have overestimated antisense transcription [84]. A thorough comparison of different strand-specific RNA-seq protocols indicated that the least amount of ‘false’ antisense RNA was generated when libraries were prepared by ‘dUTP second-strand marking’ or by using a so called ‘RNA-ligation method’, involving the sequential ligation of 3′-preadenylated and 5′-adapters to RNA [85]; protocols for both approaches have been published for *P. falciparum* [31•, 86]. Thus, a comprehensive NGS toolbox is now available to fully explore the role of ncRNAs in *Plasmodium*.

### Telomeric and subtelomeric lncRNAs

Subtelomeres of most eukaryotes contain patchworks of genes, pseudogenes and various repeat regions that are packed into constitutive heterochromatin characterized by high levels of H3K9me3 and CpG methylation [33]. Transcription of subtelomeric genes, or genes artificially inserted into subtelomeric regions, is repressed in a position-dependent manner, a phenomenon referred to as telomere position effect (TPE). TPE-like repression has been described for organisms as divergent as humans, *Drosophila melanogaster*, *Saccharomyces cerevisiae* and *P. falciparum* [18, 34, 35, 36]. Intriguingly, in 2007, telomeric repeat containing lncRNAs (TERRA), RNA molecules transcribed by RNA pol II from the subtelomere toward the chromosome end in a strand-specific manner, were discovered in humans [37]. TERRA-like transcripts have now been identified in numerous organisms including birds, *Arabidopsis*, *S. cerevisiae* and recently, in *P. falciparum* [28•, 29•, 38]. Findings from *S. cerevisiae* and human cells indicate that TERRA transcription negatively affects telomere length and genomic stability, while telomere elongation represses TERRA transcription and leads to increased levels of telomeric H3K9me3 suggesting an equilibrium between TPE and TERRA transcription [38].

**Figure 1:**
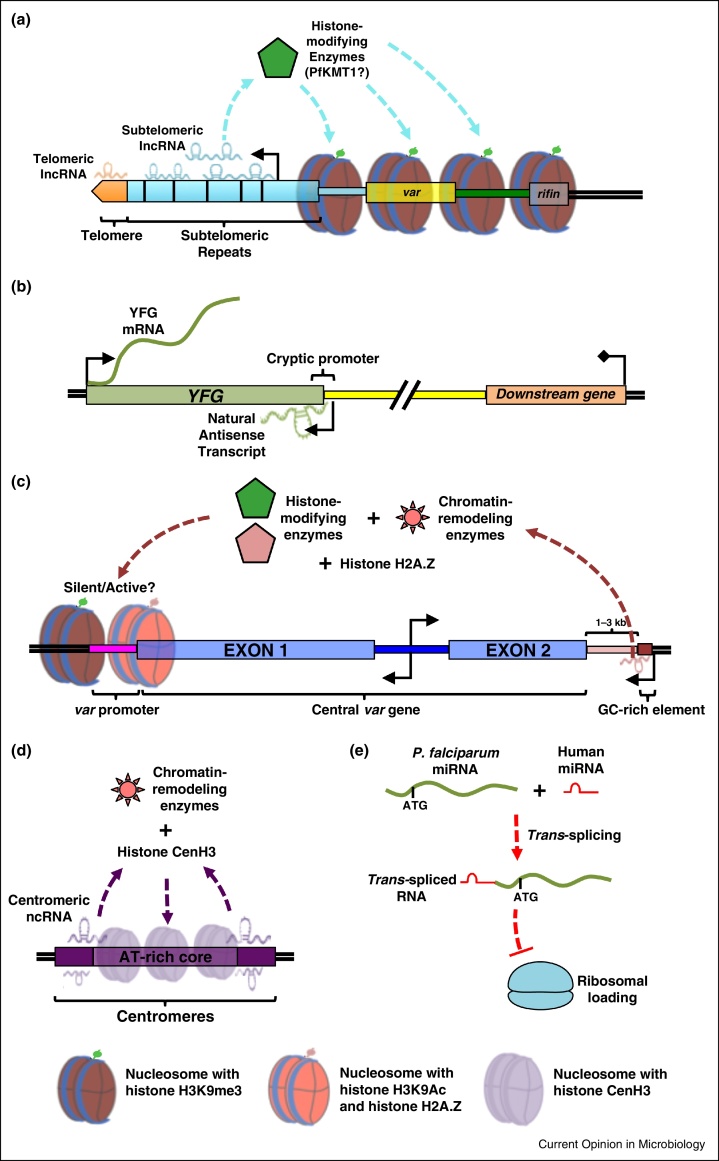
Putative functions for ncRNAs in *P. falciparum*. **(A)** Subtelomeric ncRNAs may recruit histone-modifying enzymes such as PfKMT1 to generate heterochromatin (*i.e.*, the histone H3K9me3 mark) and silence virulence gene families such as *var* and *rifin* that are proximal to the subtelomere. **(B)** A substantial number of NATs, in this case, for the gene *YFG*, are transcribed in a manner that is independent of the downstream gene, suggesting the presence of a cryptic promoter in the 3′UTR of *YFG*. **(C)** For central *var* genes, ncRNA from a proximal GC-rich element may recruit histone-modifying enzymes and/or chromatin-remodeling enzymes and determine either silencing or activation of the corresponding *var* gene. The proposed model does not take into account the TPE and nuclear clustering of *var* genes. **(D)** Centromeric ncRNAs may recruit chromatin-remodeling enzymes to place histone CenH3 at centromeres. **(E)** During blood stages, human miRNAs translocate into the cytoplasm of the parasite and are *trans*-spliced to select essential mRNAs (mechanism unknown). The resulting *trans*-spliced RNA is blocked for ribosomal loading, and hence translation.

In *P. falciparum*, using microarrays and rapid amplification of cDNA ends (RACE), Broadbent *et al.* identified 1.5 kb and 3.1 kb long transcripts from at least 15 of the 28 chromosome ends, with transcript levels peaking 40 h post-erythrocyte invasion (pi) [28^•^]. In contrast, Sierra-Miranda *et al.* identified longer TERRA-like transcripts, ∼4 kb and >6 kb long, in multiple *P. falciparum* blood stages [29^•^]. Using RNA-fluorescence *in situ* hybridization (FISH), they showed that these subtelomeric transcripts form multiple nuclear foci during schizogony, and cluster in a unique perinuclear locus in early blood stages, which is distinct from other subnuclear compartments (such as telomeric clusters and ribosomal DNA clusters) [29^•^]. In fact, we, along with others, observed pervasive telomere-directed transcription of subtelomeres in *P. falciparum in vitro* culture (Siegel and Scherf, unpublished observations) [27, 28•]; however, the biological significance of this class of ncRNA remains elusive. One observation suggests a role for TERRA-like transcripts in maintaining telomere ends in *P. falciparum*: chromosome breakage and healing events that delete entire subtelomeric repeat regions of *P. falciparum* chromosomes result in telomere elongation [39], possibly due to the loss of transcription of TERRA-like lncRNAs. Additionally, given the conspicuous subtelomeric location of virulence genes in *P. falciparum*, subtelomeric lncRNAs may create a default silencing environment for these genes by enabling the establishment of subtelomeric heterochromatin (Figure 1a). This may be key to regulating the clonally variant, mutually exclusive expression of virulence genes.

### Natural antisense RNA transcripts

NATs exist in all kingdoms and are often highly abundant; for example, in mice, NATs have been identified for more than 70% of transcription units [40]. In addition, many NATs are highly conserved and expressed in a developmentally regulated manner, with tissue-specific expression patterns. The mechanisms of NAT-mediated regulation of gene expression, which can be positive or negative, have been grouped into four general categories: transcriptional interference, nuclear RNA–RNA duplex formation, cytoplasmic RNA–RNA duplex formation, and RNA–DNA interactions (see Box 2 for more details) [41]. Importantly, all of these mechanisms can mediate gene expression in a dicer-independent manner and may be of particular importance in organisms lacking a functional RNAi pathway like *P. falciparum*.

Box 2 **Mechanisms of NAT-mediated gene regulation**

Natural antisense transcripts (NATs) can regulate gene expression in a positive or negative manner and these mechanisms have been grouped into four categories [41]:

(1) *Transcriptional interference*: The act of antisense transcription leads to the collision of RNA polymerases, thus interfering with sense transcription [87, 88, 89]; the NAT itself does not affect gene expression.
(2) *Nuclear RNA–RNA duplex formation*: The formation of sense RNA–antisense RNA duplexes in the nucleus affects RNA splicing, editing, and mRNA export [90, 91, 92].
(3) *Cytoplasmic RNA–RNA duplex formation*: The formation of cytoplasmic sense RNA–antisense RNA duplexes stabilizes or destabilizes mRNA transcripts, or affects translation without altering mRNA stability [93].
(4) *RNA–DNA interactions*: In contrast to transcription factors, DNA methyltransferases, histone-modifying enzymes, and chromatin remodeling factors generally lack specific DNA-binding motifs. Yet, they are capable of placing epigenetic marks at specific genomic loci, permitting localized regulation of expression. The observed specificity may be mediated by NATs that form specific RNA–DNA interactions while at the same time binding to chromatin modifying enzymes. NATs have been shown to affect various forms of epigenetic regulation, including DNA methylation [94], chromatin modifications [95] and monoallelic expression (*e.g.*, genomic imprinting [96] and X chromosome inactivation [97]).

Similar to *S. cerevisiae* and mammals, NATs have been observed in *P. falciparum* [22, 23, 30•, 31•, 32•] and their occurrence seems pervasive, with a conservative estimate of NATs being transcribed from 24% of all open reading frames (ORFs) [31^•^]. NAT levels are highest toward the 3′ end of the ORF and for some of the NATs, their levels significantly correlate with transcript levels of the gene located downstream: this suggests that NATs may be the result of incomplete transcription termination or bidirectional transcription initiation [31^•^]. However, for the majority of NATs, no such dependency is observed, suggesting the presence of cryptic promoters that drive their transcription [31^•^]. While functional studies are missing in *P. falciparum*, sense and antisense RNA levels as well as antisense RNA and protein levels appear to be uncorrelated [30•, 31•]. This indicates that *P. falciparum* NATs most likely do not function through transcriptional interference, or RNA–RNA duplex formation, but may regulate the *P. falciparum* epigenome. Given the highly defined epigenetic landscape in *P. falciparum* (see also the review by Voss *et al.*, in this issue), it will be interesting to see if future research reveals a link between NATs and the recruitment of factors that modify the parasite epigenome (Figure 1b).

### Noncoding RNAs and the regulation of virulence genes

The major virulence factor of *P. falciparum* blood stages, PfEMP1 (Erythrocyte Membrane Protein 1), is an exported surface protein that mediates cytoadhesion and immune evasion, events that are essential for malaria pathogenicity. PfEMP1 is encoded by the ∼60 member *var* gene family and is subject to antigenic variation by mutually exclusive gene expression [11]: a single *var* gene is actively transcribed 8–20 h post-erythrocyte invasion (pi) [42] and remains in a transcription-ready or poised state thereafter [43], while the remaining *var* genes are transcriptionally inactive throughout the IED.

**Figure 2:**
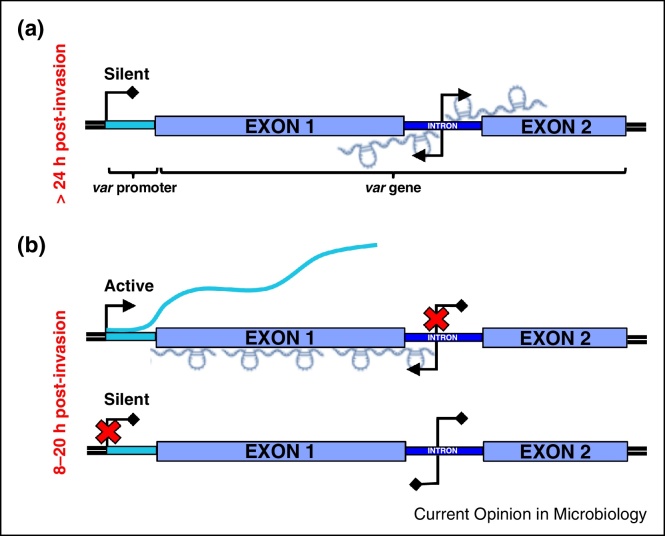
lncRNA transcription from the introns of *var* genes is regulated in a stage-specific manner. **(A)** In late stages, that is, >24 h pi, sense and antisense lncRNAs are generated from silent *var* genes. **(B)** In the ring stages, that is, 8–20 h pi, transcription of the active *var* gene is accompanied by antisense lncRNA production from the intronic promoter. There is no lncRNA generated for silent *var* genes.

Each *var* gene is composed of a variable exon 1 and a conserved exon 2 that are separated by a conserved intron [44, 45, 46, 47]. In 1995, Su *et al.* detected transcripts of 1.8–2.4 kb in *P. falciparum* mixed blood stage cultures that mapped to the intron–exon 2 junctions of several *var* genes; these were termed ‘sterile’ transcripts [46]. Later, it was demonstrated that these ‘sterile’ transcripts resulted from promoter activity within the *var* intron [47] and that transcription occurs at the onset of DNA replication (∼24 h pi) and continues through schizogony and merozoite formation [42]. Next, Deitsch and colleagues showed that the A-rich central region of *var* introns has bidirectional promoter activity and generates antisense and sense lncRNAs that extend into *var* exon 1 and 2, respectively [48] (Figure 2a). Using strand-specific RNA-FISH and ChIP, they found that these lncRNAs localize to discrete perinuclear foci and remain associated with chromatin. Although the authors were unable to attribute function, they suggested that intron promoter activity and intronic lncRNAs are linked to silencing of all *var* genes in late stages of the IED. In contrast, two other studies found intronic antisense lncRNAs associated with the single active *var* gene in early stages of the IED, that is, 8–20 h pi [49, 50] (Figure 2b). Whether the antisense lncRNAs transcribed from the active *var* intron at different stages of the IED are identical in sequence and serve the same purpose remains to be seen.

From these studies, the intron has been hypothesized to play a pivotal role in regulating *var* expression. However, it is not clear if it is transcription from the intron or the resulting lncRNAs that play(s) a role. Firstly, even though intronic lncRNAs associate with chromatin, the exact nature of this interaction is unclear. Genome-wide studies showed that the nucleosome landscape of *var* introns changes during IED, that is, from relatively loose packing up to 36 h pi to rapid compaction at 36 h pi [51]. Given that loading nucleosomes onto DNA can be directed by lncRNA in conjunction with chromatin-remodeling enzymes [52], a similar process may occur in *P. falciparum var* introns. Secondly, intronic transcription is observed for both the poised and silent *var* genes at late stages of IED [48], suggesting that the effect of the intronic lncRNAs on *var* expression is most likely multifold. Recently, the human lncRNA lincRNA-Cox2 was shown to activate and repress different sets of genes through interaction with specific heterogeneous nuclear ribonucleoproteins (hnRNPs) [53], while *S. cerevisiae GAL* lncRNAs were suggested to maintain the inducible *GAL* genes in a poised state and enable rapid RNA polymerase II recruitment [54]; the *var* intronic lncRNAs could play similar roles [55]. Thirdly, in addition to specific histone H3 modifications (see Introduction), the promoters of active *var* genes associate with nucleosomes containing the histone H2A variant H2A.Z [56, 57]. The intronic lncRNAs could be responsible for this nucleosome-remodeling event as well as the recruitment of histone-modifying enzymes to the *var* promoter [55]. Taken together, the contribution of the intron to singular *var* choice is still unclear.

While most *var* genes are found within subtelomeric clusters, ∼30% of them are found in internal chromosomal regions and are referred to as ‘central’ *var* genes. The intergenic regions of the central *var*s contain highly conserved (89.9–100% homology) GC-rich (35–40%) sequence elements. Fifteen such GC-rich elements have been identified in the *P. falciparum* genome [58, 59, 60], and given their positioning and sequence conservation, it is hypothesized that they may play a role in regulating *var* expression. Intriguingly, microarray analysis revealed transcripts for three of the 15 elements (PF3D7_0421000, PF3D7_0712100, PF3D7_1241000) [26], with directionality of transcription opposite from and toward the *var* gene (Figure 1c). However, rigorous analysis of the GC-rich elements at the transcriptional level has been difficult due to their high degree of sequence similarity. One speculation is that ncRNA corresponding to the GC-rich element may dictate singular *var* choice in an epigenetic manner (Figure 1c) [61^•^].

### Other ncRNAs

Centromeric ncRNAs were the first chromatin-associated ncRNAs to be described in *P. falciparum* blood stages [62]. Similar to the *var* intronic promoter, the centromeric promoter was shown to possess bidirectional activity, generating two small RNAs, 75 and 175 nt long, that localize to the nucleus and associate with centromeric DNA *via* interaction with an unknown protein. At that time, the authors suggested that the centromeric ncRNA is essential to forming heterochromatin at *P. falciparum* centromeres. It is now evident that the histone H3 variant, PfCenH3, and H2A.Z demarcate *P. falciparum* centromeres; however, they lack heterochromatin hallmarks like H3K9me3 that are found at centromeres of other eukaryotes [63, 64]. It remains to be seen if the centromeric ncRNA acts as a tether to recruit PfCenH3 to AT-rich *P. falciparum* centromeric sequences (Figure 1d).

Although parasite-derived miRNAs, a class of small RNAs generated by the RNAi pathway, have not been identified [65], host miRNAs appear to be present in parasite RNA preparations [66]. A recent study compared the miRNA composition of sickle cell and normal erythrocytes and found that two miRNAs, miR-451 and let-7i, were upregulated in sickle cell erythrocytes [67^••^]. The authors then showed that these miRNAs translocate into the parasite, are *trans*-spliced to several essential mRNAs at the 5′ end, and abolish their translation by preventing ribosomal loading (Figure 1e); this highlights a possible host defense mechanism and provides one explanation for the refractoriness of sickle cell erythrocytes to *P. falciparum* infection [67^••^]. These observations also raise the question: do the host and parasite interact at the RNA level? Given the recent discovery that infected erythrocytes communicate with each other *via* exosomes and microvesicles that contain host and parasite proteins, parasite small RNAs, and host miRNAs [68••, 69••], and given that these exocytic vesicles can induce gametocyte production, it is not far-fetched to postulate a cross-talk between the host and parasite and, amongst different parasites within the same host.

## Perspectives

The discovery that eukaryotic genomes are pervasively transcribed — for example, 98% of the human genome [70], ∼85% of the *S. cerevisiae* genome [71], and ∼80% of the *P. falciparum* genome [31^•^] — has revolutionized the way we think about RNA. While the debate as to whether every transcription event serves a biological function or if some represent transcriptional noise continues, select ncRNAs have been extensively characterized and shown to regulate diverse processes: from post-transcriptional gene silencing by small RNAs to maintenance of chromatin structure and genomic imprinting mediated by lncRNAs [72]. Nonetheless, the ever-increasing repertoire of techniques to study ncRNAs remains to be adapted to *P. falciparum* and to date, not a single non-structural ncRNA has been functionally characterized in this parasite. As a result, compared to other organisms, *P. falciparum* ncRNA biology is clearly lagging behind.

When the *P. falciparum* genome was sequenced, a striking observation was the large number of RNA-binding proteins encoded by the parasite [10]. To help elucidate if some of these proteins play a role in ncRNA-mediated gene regulation, UV cross-linking immunoprecipitation (CLIP) experiments followed by high throughout sequencing [73] could be performed. In addition, this strategy could be used to determine if histone-modifying enzymes like PfSir2 and PfKMT1 are recruited to *var* genes by subtelomeric or *var* intronic lncRNAs.

With the continued increase in ncRNA number and diversity, several groups have reported that select putative ncRNAs encode for proteins and important regulatory peptides as small as 11 aa in length [74]. Thus, a thorough characterization of the *P. falciparum* noncoding transcriptome will require a comprehensive delineation of the parasite's translatome, using, for example, the recently developed ribosome-profiling technique, which can be used to map the translatome at subcodon resolution [75]. Ribosome profiling has been successfully used to differentiate coding RNA from ncRNA in mammals and other protozoan parasites [76, 77] and should be transferable to *P. falciparum*.

Lastly, functional characterization of the different ncRNAs will require techniques to selectively deplete individual transcripts in *P. falciparum* without affecting other genomic loci. Given that in just over a year of its emergence as a genome-editing tool, the CRISPR-Cas9 system has been utilized to generate insertions, gene deletions, and even single nucleotide substitutions in a selectable marker-free manner in multiple prokaryotic and eukaryotic cells [78], its adaptation to *Plasmodium* should have tremendous potential. It should enable the deletion of specific *var* introns or even GC-rich elements and aid in understanding their contribution to mutually exclusive *var* expression.

## References and recommended reading

Papers of particular interest, published within the period of review, have been highlighted as:

• of special interest
•• of outstanding interest
